# The Impact of DNA Methylation Dynamics on the Mutation Rate During Human Germline Development

**DOI:** 10.1534/g3.120.401511

**Published:** 2020-07-29

**Authors:** Yijia Zhou, Funan He, Weilin Pu, Xun Gu, Jiucun Wang, Zhixi Su

**Affiliations:** * Ministry of Education Key Laboratory of Contemporary Anthropology, School of Life Sciences, Fudan University, Shanghai, 200433, China; † Department of Genetics, Development and Cell Biology, Iowa State University, Ames, IA 50011; ‡ Department of Anthropology and Human Genetics, School of Life Sciences, Fudan University, Shanghai, 200433, China; § Human Phenome Institute, Fudan University, Shanghai, 201203, China; ** Singlera Genomics Inc., Shanghai, 201318, China

**Keywords:** DNA methylation, 1000 Genome Project, germline mutation rate, early fetal development, germ cell

## Abstract

DNA methylation is a dynamic epigenetic modification found in most eukaryotic genomes. It is known to lead to a high CpG to TpG mutation rate. However, the relationship between the methylation dynamics in germline development and the germline mutation rate remains unexplored. In this study, we used whole genome bisulfite sequencing (WGBS) data of cells at 13 stages of human germline development and rare variants from the 1000 Genome Project as proxies for germline mutations to investigate the correlation between dynamic methylation levels and germline mutation rates at different scales. At the single-site level, we found a significant correlation between methylation and the germline point mutation rate at CpG sites during germline developmental stages. Then we explored the mutability of methylation dynamics in all stages. Our results also showed a broad correlation between the regional methylation level and the rate of C > T mutation at CpG sites in all genomic regions, especially in intronic regions; a similar link was also seen at all chromosomal levels. Our findings indicate that the dynamic DNA methylome during human germline development has a broader mutational impact than is commonly assumed.

Germline mutations, the random genetic errors that occur in germ cells (Campbell and Eichler 2013), are the source of heritable diseases and evolutionary adaptations (Ségurel *et al.* 2014). Understanding the rate of germline mutation occurrence and the mechanisms that control it, particularly in humans, is of great importance to finding causes of heritable diseases and evidence for evolution (Chen *et al.* 2017). The mutation rate is a reflection of the dynamics of organisms. Recent studies of germline mutations have identified several factors that influence point mutation rates, including expression level, replication timing, GC content (Hodgkinson and Eyre-Walker 2011; Michaelson *et al.* 2012; Park *et al.* 2012; Francioli *et al.* 2015; Besenbacher *et al.* 2016; Goldmann *et al.* 2016) and CpG methylation (Kong *et al.* 2012). However, the origin of many mutations during human germline development remains unknown.

CpG dinucleotides are frequently methylated in the human genome. When a methylated CpG is changed into a TpG through spontaneous deamination and is not corrected by the repair system, the result is a mutation (Kong *et al.* 2012). The spontaneous deamination rate of a methylated cytosine is usually 2.0 to 3.2 times higher than that of an unmethylated cytosine (Ehrlich *et al.* 1986; Zhao and Jiang 2007; Xia *et al.* 2012). Next-generation sequencing technology and *in vitro* culturing technology are quickly developing, and whole-genome bisulfite sequencing enables researchers to obtain the dynamic DNA methylome in cells at different stages (Guo *et al.* 2015; Poulos *et al.* 2017; Prada-Arismendy *et al.* 2017; Li *et al.* 2018). In many genomics studies of cancer, the methylation-mutation association is studied across cancer types and at different disease stage checkpoints, including screening, diagnosis, prognosis and monitoring (Poulos *et al.* 2017; Prada-Arismendy *et al.* 2017; Takane *et al.* 2017; Chakravarthy *et al.* 2018). The DNA methylome undergoes extensive reprogramming during human germ cell development, especially in primordial germ cells (PGC) (von Meyenn and Reik 2015). However, how the dynamic DNA methylome during human germ cell development causes a different germline mutation rate remains poorly understood.

The methods used in researching somatic mutations have proven difficult to apply to the germline (Agarwal and Przeworski 2019). Part of the difficulty is that germline mutations occur very rarely; there are approximately 70 *de novo* mutations per generation (Jónsson *et al.* 2017). Until recently, the most direct approach for estimating germline point mutations is based on resequencing whole genomes from blood samples of human pedigrees and identifying germline mutations through comparisons of parents and offspring (Conrad *et al.* 2011; Michaelson *et al.* 2012; Campbell and Eichler 2013; Ségurel *et al.* 2014; Shendure and Akey 2015; Goldmann *et al.* 2016). However, the analysis of this method is technically challenging and is poor for identifying determinants of rate mutation factors (Chen *et al.* 2017; Agarwal and Przeworski 2019). For instance, the correlation analysis between genome features and mutation patterns combining the three largest *de novo* mutation studies produced inconsistent patterns, but the cause for this was unknown (Chen *et al.* 2017; Smith *et al.* 2018). Another way to estimate the mutation rate utilizes the divergence among species indirectly. By comparing the genomes of humans, chimpanzees, and gorillas, human mutations that differ from the most recent ancestor could be identified. This method meets the needs of large-scale analysis, but the result is highly affected by natural selection (Hodgkinson and Eyre-Walker 2011; Scally *et al.* 2012; Xia *et al.* 2012). A previous study applied human-chimpanzee divergence as germline mutations to investigate methylation-mutation association, but the authors applied the methylome only from human embryonic stem cells instead of from human germ cells. The authors claimed a high mutation rate in 20–60% DNA methylation level while highly methylated CpGs leads to a low mutation rate (Xia *et al.* 2012).

A good method for overcoming the limitation of the small density of germline mutation variants is to use polymorphisms on behalf of *de novo* mutations (Agarwal and Przeworski 2019; Kusmartsev *et al.* 2020). In large-scale mutation datasets, polymorphisms with low frequency variants are rare, so these mutations can be regarded as neutral mutations that minimize the effects of indirect selection and biased gene conversion (Schaibley *et al.* 1984; Rahbari *et al.* 2016; Carlson *et al.* 2018; Agarwal and Przeworski 2019). Thus, rare variants across large-scale populations can then be applied to investigate their associations with genomic features. Using this strategy, a recent study of low frequency variants in gnomAD (Lek *et al.* 2016) on the human X chromosome and autosomes ascribed specific mutation signatures to recombination and replication timing. They also identified differences in mutation types resulting from sex effects (Agarwal and Przeworski 2019). Another recent study of human autosomal variants identified types of mutations and genomic contexts that were strongly associated with numerous genomic features, including GC content, DNase hypersensitivity, and histone modification (Carlson *et al.* 2018). However, neither study considered the DNA methylation level.

Here, we used the whole genome bisulfite sequencing methylome of cells at 13 stages during human germline development and rare variants from the 1000 Genome Project as germline mutations to investigate the correlation between dynamic methylation level and germline mutation rate. We found a significant correlation between the methylation level and the germline mutation rate at CpG sites using a single-site resolution. We also explored the mutability of methylation dynamics in all stages at single-site resolution and found the sperm stage may be of great importance. Our results also showed a high correlation between the regional methylation level and the mutation rate of C > T at CpG sites in intronic regions during the early developmental stage. We observed indirect evidence that correlation of methylation and mutation was more significant in germ tissues than in somatic tissues. Our study provides evidence that DNA methylation in germline relevant cells has a more significant impact on CpG mutation rate than is commonly assumed.

## Materials and methods

### Selection of SNPs

All SNPs were from the 1000 Genomes Project phase 3 (http://hgdownload.cse.ucsc.edu/gbdb/hg19/1000Genomes/phase3/). We defined autosomal SNPs with allele frequencies of less than 0.1% as rare variants, which were recognized as germline mutations. To assess the quality of variants, we only included variants with a variant quality score of 100. After removing the structural variants, indels, multi-allelic SNPs and non-autosomal SNPs, we were left with 51,739,442 SNPs. We classified mutations into 9 types: CpG C > T (a C > T or G > A mutation at a CpG site), CpG C > A (C > A or G > T at CpG), CpG C > G (C > G or G > C at a CpG site), non-CpG C > T (a C > T or G > A mutation at a non-CpG site (CHH or CHG)), non-CpG C > A (C > A or G > T at a non-CpG site), non-CpG C > G (C > G or G > C at a non-CpG site), non-CpG T > C (T > C or A > G at a non-CpG site), non-CpG T > G (T > G or A > C at a non-CpG site) and non-CpG T > A (T > A or A > T at a non-CpG site). The distribution of each SNP type in the human genome is shown in Figure S1. The proportion of each SNP type is calculated as the ratio of the number of the specific type of SNP out of the total number of SNPs. The distribution of all types of mutations in different genomic regions is shown in Figure S1B.

### Estimation of mutation rate

We estimated the mutation rate using SNP density for different mutation types at both site-level and regional-level analyses.

### Site-level analysis

For mutation rates at single CpG sites, we used SNP density to represent the possibility of SNP occurrence. For each cell stage, the SNP density was estimated as the ratio of the total number of CpG C > T, CpG C > G and CpG C > A SNPs out of the total number of covered sites within a specific range of methylation levels.

### Pattern-level analysis

We also applied SNP density to estimate mutability of certain dynamic methylation pattern at a single site. For each pattern, the SNP density was computed as the ratio of total number of CpG SNPs out of the total number of that methylation pattern. Only patterns with CpG SNP number ≥10 are included in the ranking.

### Regional-level analysis

We used 2 measurements to test the effect of the methylation level on mutation at CpG or non-CpG sites: CpG C > T density and non-CpG C > T density. The calculation of these 2 measurements and their representation were performed as follows:

1. CpG C > T density was quantified by the ratio of the number of CpG C > T SNPs in a window to the total number of C and G on CpG sites in the window, which revealed the C > T mutation rate on CpG sites.
2. Non-CpG C > T density was calculated by the ratio of the number of C > T and G > A mutations on non-CpG sites in a window to the number of C and G on non-CpG sites in the window, which revealed the mutation rate for the C > T SNP on non-CpG sites.

For different genomic regions, we chose 1 kb as the window size.

For chromosomal calculations, we chose a 1 MB window size.

For promoter regional CpG mutation rate, CpG density was quantified by the ratio of the number of CpG SNPs in a promoter region to the total number of C and G on CpG sites in the promoter region.

All analyses were performed by applying customized python, R statistical packages and bedtools (https://bedtools.readthedocs.io/en/latest/).

### Analysis of DNA methylation data during germline development

The processed whole-genome bisulfite sequencing (WGBS) DNA methylation data from sperms, oocytes, 8-cell stage embryos, morulae and ICM cells were kindly provided by Li *et al.* (2018) (Li *et al.* 2018). The processed WGBS DNA methylation data of primordial germ cells from 7-week-old males, 10-week-old males and females, 11-week-old males and females, 13-week-old males, 17-week-old females and 19-week-old males, were downloaded from Guo *et al.* (2015) (Guo *et al.* 2015).

### Site-level analysis

The methylation level for single sites was calculated as the ratio of methylated reads (*i.e.*, reads with a C at this site) out of the total number of covered reads (*i.e.*, reads with a C or T at this site) at the same reference position. Each site covering at least 5 reads was taken into calculation. In addition, we used a binomial test to identify the methylated sites. According to the single-site methylation level, methylated CpG sites were further categorized into five groups: methylation levels of 80–100%, 60–80%, 40–60%, 20–40% and 0–20%.

To avoid bias caused by the different coverage of different cell stage samples, we selected all common sites covering at least 5 reads from all cell stages. In total, we identified 561,800 sites covered in all cell samples. Apart from methylated CpG sites and unmethylated CpG sites, the single-site methylation level was also categorized into five groups: methylation levels of 80–100%, 60–80%, 40–60%, 20–40% and 0–20%.

### Pattern-level analysis

We would like to explore the relationship of methylation dynamics with mutation rate at one single site. To illustrate the dynamic methylation patterns for single sites in 13 stages, we classified methylation level into three groups: highly methylated (methylation level ≥70%), Methylated (70% > methylation level ≥20%) and unmethylated (methylation level < 20%). In total, we found 27,735 unique patterns for single sites.

### Regional-level analysis

For different genomic region analyses, we binned all the genomic regions into 1-kb tiles. Only tiles containing at least 5 CpGs were considered for further calculation. The DNA methylation level of each tile was quantified by the weighted methylation level as the fraction of methylated C reads divided by the total number of C and T reads in this tile.

For chromosomal calculation, we chose a 1-MB window size. The average DNA methylation level for chromosomes was also estimated as C: (C+T) ratios. All analyses were performed by applying customized python, R statistical packages and bedtools. Note that all the DNA methylation levels mentioned in this study are specifically referred to as the DNA methylation levels at CpG sites.

### Multiple linear regression

We performed multiple linear regression to estimate the impact of methylation dynamics in each stage on CpG mutation. We normalized the mutation for each single site as 0 for unmutated and 1 for mutated. The methylation level of each site in all stages is derived from single-site methylation level. The linear model was based on the common sites covered in all samples (details see Table S3). We then constructed the linear model in a stepwise fashion using R statistical packages to explore the most important stages.

### Analysis of human gene expression data

We used the processed expression data from 409 microarray experiments in our previous study (Su *et al.* 2011b), which were obtained from Graham McVicker *et al.* (McVicker and Green 2010). Graham McVicker collected 409 samples of expression data from several studies (Sato *et al.* 2003; Su *et al.* 2004; Barberi *et al.* 2005; Ge *et al.* 2005; Skottman *et al.* 2005; Looijenga *et al.* 2006; Korkola *et al.* 2006; Kocabas *et al.* 2006; Houmard *et al.* 2009; Wu *et al.* 2009), representing a wide range of germ and somatic tissues. The methods to process the raw data were described in Graham McVicker *et al.* (McVicker and Green 2010). We also included expression data of 15 primordial germ cell stages and 10 somatic cell stages during early development, which were published by Guo *et al.* (2015) (Guo *et al.* 2015). The expression data of 6 cell stages during spermatogenesis were obtained from Jingtao Guo *et al.* (Jan *et al.* 2017). The single cell expression data from 2 spermatogonial stem cells were obtained from GSE92276 (Guo *et al.* 2017). The source and classification of all the expression data are described in detail in Table S5. In total, we assigned an expression intensity to 7707 genes in 442 tissues for SNP density analysis and 7794 genes for CpG_O/E_ ratio analysis. Among the 442 tissues, 79 contained germ cells and were considered germline tissues.

### Calculation of the observed over expected CpG ratio (CpG_O/E_)

The observed over expected CpG ratio was calculated as the ratio of the frequency of CpG over the frequency of C and G (Elango *et al.* 2009). We used CpG_O/E_ to infer the germline mutation rate at CpG sites in the promoter region (in a range of -1000 to +200 bp from the transcription start site). CpG_O/E_ is a robust measure of the germline mutation rate on CpGs on an evolutionary time scale. The accumulation of inheritable mutations on CpGs over evolutionary time is somewhat reflective of CpG_O/E_. Consequently, genomic regions with a high cytosine germline mutation rate have lower-than-expected CpG_O/E_. In contrast, regions with low mutation rates maintain a high CpG_O/E_. CpG_O/E_ is an indirect measure of the historical CpG mutation rate.

### Genome sequence annotation

All the datasets were based on the hg19 (GRch37) reference. Genomic annotations, including the start and end positions of genes, exons, enhancers, CpG islands (CGIs), introns and repeat regions, were derived from UCSC Genome Browser tracks (http://genome.ucsc.edu). The promoter was defined as an interval of -1000 to +200 bp around the transcription start site (TSS), as previously described (Su *et al.* 2011b). An intergenic region was defined as a region not defined by other regions. An intragenic region here was referred to as the gene region. All kinds of repeated regions were excluded from other regions. We also eliminated the common region of the promoter-enhancer and CGI-enhancer from the enhancer region.

### Data availability

Table S1 shows the correlation between the mutation rate of methylated and unmethylated CpG sites in all stages for all sites and Table S2 for common sites. Table S3 shows the methylation level and SNP state for common sites in all stages. Table S4 displays the mutability of dynamic methylation patterns for single sites. Table S5 shows the type and source of expression data for 442 samples.

Figure S1 shows the distribution of SNPs for each type of mutation in the human genome. Figure S2 illustrates the mutation rates for all CpG sites during developmental stages. Figure S3 displays the Spearman correlations between gene expression and the CpG_O/E_ ratio in the promoter regions for 7 tissue types. Figure S4 shows the distribution of methylation level and CpG C > T density of 1-kb windows in different genomic regions. Figure S5 illustrates the Spearman correlation between methylation and C > T mutation rates on CpG and Non-CpG sites in different chromosomes during germline development.

Other sources of public data we applied were described in the corresponding section of Materials and Methods in detail. Supplemental material available at figshare: https://doi.org/10.25387/g3.12652865.

## Results

### Single-site methylation levels are positively associated with mutation rates at CpG sites during different developmental stages

To investigate methylation-mutation correlation at the single site level, we measured the mutation rate using the SNP density on CpG sites within different methylation levels during different developmental stages. We would like to compare mutation rates at methylated or unmethylated sites, as well as sites with different methylation levels. To explore large-scale germline variants in humans, we chose 51,739,442 singleton autosomal SNPs (allele frequency < 0.1%) filtered from the 1000 Genomes Project as a proxy (Figure S1). For each cell stage, we estimated the mutation rate at CpG sites by computing the number of CpG C > T, CpG C > G and CpG C > A SNPs over the number of covered sites within the same methylation level group. For each cell stage, all the covered CpG sites were classified into three groups, total CpGs, methylated CpGs and unmethylated CpGs. As for methylated CpG sites, we further equally divided them into 5 groups according to their methylation levels.

As expected, we found that methylated CpGs were more likely to mutate compared with the unmethylated (Figure S2). Taking the sperm stage as an example, unmethylated CpGs had a much lower mutation rate (4.92%) than methylated CpGs (17.73%) (chi-squared test, *P* < 2.2E-16). Among the five DNA methylation level categories in sperm, we found a trend that when the CpG methylation level increased, the mutation rate increased as well (Pearson’s r = 0.985). A high correlation between methylation level and mutation rate was also observed in the other 12 stages of cells (Figure S2, Table S1).

**Figure 1:**
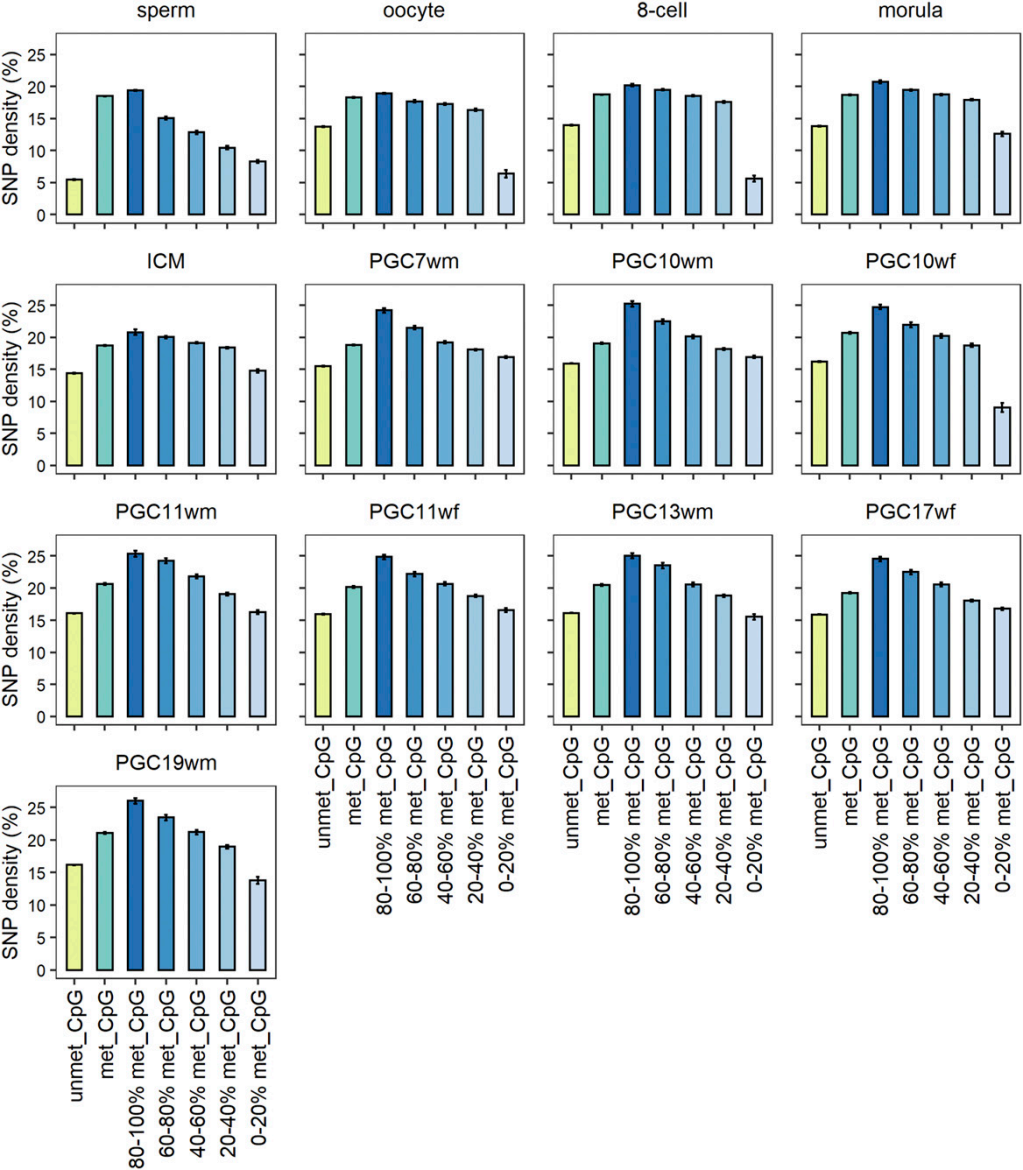
The mutation rates for all CpG sites, unmethylated CpG sites and methylated CpG sites during developmental stages. The single-site methylated CpG levels are further classified into five different DNA methylation level categories (80–100%, 60–80%, 40–60%, 20–40%, and 0–20%). The Y-axis shows the mutation rate computed by the SNP density at CpG sites.

To avoid sequencing coverage bias of different cell samples, we chose common CpG sites in all cell stages covered at least 5 reads, including 56,100 CpG sites in total (Table S3). We could observe subtle differences in the relationship of methylation with mutation rate within stages based on common sites. We found CpG methylation levels positively correlates with mutation rates in all cell stages (Figure 1). At the sperm stage, Methylated CpGs had a significantly high mutation rate (18.50%) than the unmethylated (5.44%) (chi-squared test, *P* < 2.2E-16). We also observed a positive correlation between CpG mutation rate and the 5 methylation levels in sperm stage (Pearson’s r = 0.989, *P* = 0.001), as well as in other 12 stages (Figure 1, Table S2). Among 13 cell stages, we found subtle difference in correlation between methylation and mutation. For example, the correlation rho of sperm and PGC stages are a little higher than that of early embryo stages (Table S2). This phenomenon suggests that methylation in all the stage may be the source of some CpG mutation, but their contributions may differ.

On the other hand, we observed that unmethylated CpGs had a slightly higher mutation rate than the least methylated CpGs (0–20%) in oocyte, 8-cell embryo, morula, PGC10wf (primordial germ cells from 10-week-old females), PGC11wm (primordial germ cells from 11-week-old males), PGC13wm and PGC19wm stages. We assume this was because we filtered methylated CpGs with a strict binomial test, as mentioned in the Materials and Methods section. Many CpG sites with extremely low methylation levels and low quality were characterized as unmethylated sites, which in turn increased the mutation rate of unmethylated CpG sites.

Interestingly, our results are obviously different from the results of another study by Junfeng Xia *et al.* (2012) (Xia *et al.* 2012). They found a high mutation rate on 40–60% and 20–40% CpG methylation levels instead of highly methylated CpG sites using the methylome of ^1^H embryonic stem cells and human-chimpanzee divergence to identify germline mutations. We think that the different results partly stem from the differences in germline mutation sources. Another reason lies in that the methylome of ^1^H embryonic stem cell represents methylation level for somatic cells instead of germline cells at the same stage.

### Mutability of methylation dynamics during germline development

Since we found subtle differences among stages (Figure 1), we would like to investigate in which stage methylation contributes more to mutations. Methylation level at a certain site may change dramatically during early fetal development (Guo *et al.* 2014, 2015). To illustrate the methylation dynamics across stages, we classified methylation level into three groups, highly methylated (methylation level ≥70%), unmethylated (methylation level < 20%) and methylated (methylation level in between) (Kusmartsev *et al.* 2020). We then calculated the mutation rates for different combinations of methylation level groups on a certain site in all the stages. The combinations of highly methylated/methylated/unmethylated groups in all stages are named dynamic methylation patterns in this manuscript. The mutability of a certain dynamic methylation pattern is estimated by the CpG SNP density (SNP number ≥ 10).

**Figure 2:**
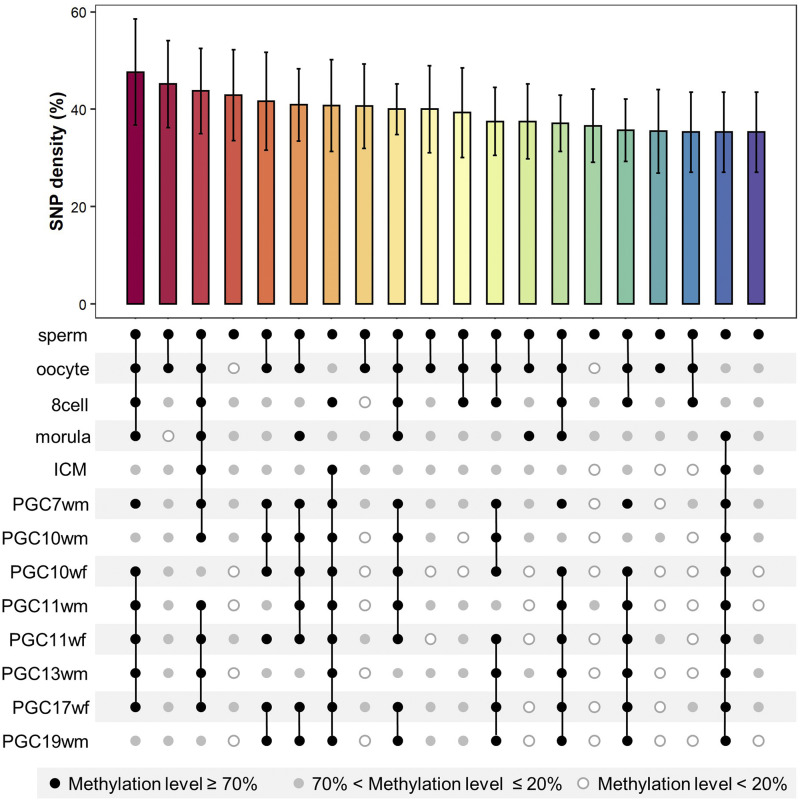
The most mutable methylation dynamic patterns during germline development. The top histogram shows the top 20 mutation rate of all methylation patterns at a single site, computed by SNP density. The bottom plot shows the methylation dynamics of one common site in 13 stages. The black dot represents that the site is highly methylated (methylation level ≥ 70%), the white dot represents unmethylated (methylation level < 20%) and the gray dot represents methylation level in between.

We showed the dynamic methylation patterns with top 20 mutation rates (Figure 2). Among the top 20 mutated patterns, we observed that the sperm stage was all highly methylated. We assume it not only results from that sperm stage stays over 80% methylation level in the whole genome scale, but also reveals a higher correlation of methylation in sperm stage with mutation. Other mutation rates of dynamic methylation patterns see Table S4.

The dynamic methylation patterns illustrated the methylation state of one certain site in all the stages, but the number of patterns is so big that we could hardly estimate the impact of methylation in each stage on CpG mutation rate. We next performed multiple linear regression using raw methylation level in common sites during all stages (Table S3). The SNP on the corresponding site was stated as “1” and non-SNP as “0”. As shown in left part of Table 1, the methylation of each stage influences CpG mutation differently. The methylation level of sperm, oocyte, PGC7wm, PGC11wm, PGC11wf, PGc17wf and PGC19wm stages positively correlation with CpG mutations while that of ICM stage negatively related. The remaining stages’ methylome had little correlation with mutation. We further used stepwise regression to disentangle which stage might be the most important in determining the CpG mutation rate. Most coefficients in the stepwise model were similar as the normal regression model (Table 1). As expected, we found the sperm stage had the highest coefficient in the regression model. This result corresponded with what we observed in dynamic methylation patterns. The methylation in sperm stage may be of greater importance to the germline CpG mutation than other stages.

**Table 1:** Summary of multiple linear regression models

Stages	Estimate	S.E.	P value	Stepwise regression
sperm	0.1386	0.001562	< 2.2e-16 *^a^*	0.1387 *^a^*
oocyte	0.0076	0.001741	1.24e-05 *^a^*	0.0078 *^a^*
8cell	0.0039	0.002454	0.1146	
morula	−0.0035	0.00283	0.2141	
ICM	−0.0086	0.002997	0.0040 *^b^*	−0.0085 *^b^*
PGC7wm	0.0127	0.003719	0.0006 *^a^*	0.0138 *^a^*
PGC10wm	0.0045	0.004829	0.3491	
PGC10wf	0.0030	0.005214	0.5597	
PGC11wm	0.0169	0.006556	0.0099 *^b^*	0.0192 *^b^*
PGC11wf	0.0221	0.005125	1.60e-05 *^a^*	0.0237 *^a^*
PGC13wm	0.0012	0.005549	0.8330	
PGC17wf	0.0136	0.004822	0.0046 *^b^*	0.0151 *^b^*
PGC19wm	0.0216	0.005493	8.39e-05 *^a^*	0.0231 *^a^*

b *P* < 0.01.

a *P* < 0.001.

### Correlation of dynamic regional methylation and C > T mutation rates during germline development

We then would like to explore the differential effect of regional methylation level on mutation rate for different types of mutations among different genomic regions. We divided the genome into 7 regions: promoter, CGI, enhancer, intergenic region, intragenic region, exon, intron. The regions were organized into several 1-kb windows and then analyzed for Spearman correlation and mutation rate for different regions in different developmental stages. Here, we compared the relationship of dynamic methylation with C > T mutation rate on CpG sites and non-CpG sites. The detailed calculation used to determine the methylation level and mutation rate are described in the Materials and Methods section.

**Figure 3:**
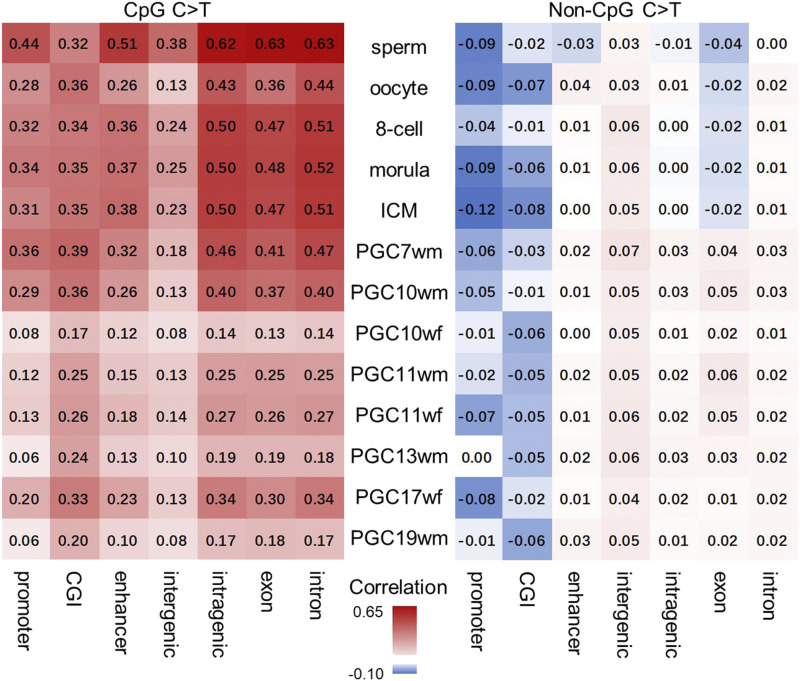
Heatmap of Spearman correlation between methylation and C > T mutation rates on CpG and Non-CpG sites in different genomic regions during germline development. The window size was 1 kb.

Correlation of methylation and CpG C > T mutation rate was more significant compared with that of methylation and non-CpG C > T mutation rate in all the genomic regions and stages (Figure 3). This result was expected, since methylated regions are more likely to have CpG C > T occurrence.

Among the different stages of cells, sperm tended to have a higher correlation of methylation and CpG C > T mutation rate in most regions. One possible explanation is that methylation levels is higher in sperm stage (greater than 80%). In addition, oocytes, the 8-cell stage embryos, morulae, ICM cells, PGC7wm cells and PGC10wm cells had more significant correlations between methylation and the CpG C > T mutation rate in most regions than the other stages.

We found that all the regions showed some amount of correlation between methylation and the CpG C > T mutation rate. The intronic region has the highest Spearman correlation of 0.63 in the sperm stage in the CpG C > T section, as well as high correlation values in other germline developmental stages. This is likely due to there being less selective pressure in the intronic region than there is in the exonic region. We expected another region with little selection, the intergenic region, to exhibit high correlation. However, we observed a relatively low correlation in this region compared with other regions. We further examined the methylation-mutation distribution of those 1-kb windows in different regions (Figure S4). A dot plot for 1-kb windows of the intergenic region showed that a high methylation level (> 0.5) had no correlation with the CpG C > T mutation rate in most cell stages (Figure S4D).

We further examined the correlation between the methylation level and the mutation rates among chromosomes. We chose a 1-Mb window and the same 2 mutation rate measurements that were used to analyze genomic regions. Compared with non-CpG C > T mutations, the CpG C > T mutation rate and methylation level had the highest correlation, as expected (Figure S5). The correlation between methylation and the C > T mutation rate was significant in all stages, except for oocytes. Sperm and all primordial germ cells exhibited a higher correlation of methylation and the CpG C > T mutation rate than the other stages. Among all the chromosomes, we observed a significant correlation between the methylation level and both 2 mutation rates on chromosome 9. In addition, chromosomes 11, 14, and 17 also showed a high correlation in the CpG C > T mutation rate. Chromosomes 6 and 20 exhibited a significant correlation between the CpG C > T mutation rate and the primordial germ cell stages. Meanwhile, chromosomes 16 and 21 showed weak correlations with the CpG C > T mutation rate. We also found that the highest correlation was on chromosome 22 between the CpG C > T mutation rate and methylation during the sperm stage.

### Indirect evidence indicates correlation between the methylation level and the CpG mutation rate in promoter regions among germline tissues

We tried to reveal methylation-mutation relationship in a life cycle for human, from one generation to the next. However, some human germ cells and tissue samples are not available because of restrictions in technology and ethics. For lack of methylome in several cell stages, especially gamete genesis stages, we tried to find a proxy for DNA methylation. Methylation of the gene promoter region has long been studied and recognized as a suppressor of gene expression (Su *et al.* 2011a; Guo *et al.* 2015). The promoter region is always one of the focus regions in methylation studies. Using expression data, we could explore to what extent methylation correlated with mutation in more embryonic tissues. Here, we used gene expression data from 442 embryonic tissues to negatively represent the corresponding methylation level of the promoter region. The 442 embryonic tissues were classified into 7 groups, including germline tissues and somatic tissues. The CpG mutation rate in promoter region is calculated as the regional SNP density (see Materials and Methods). We calculated the Spearman correlaton between CpG mutation rate and gene expression for all tissues.

**Figure 4:**
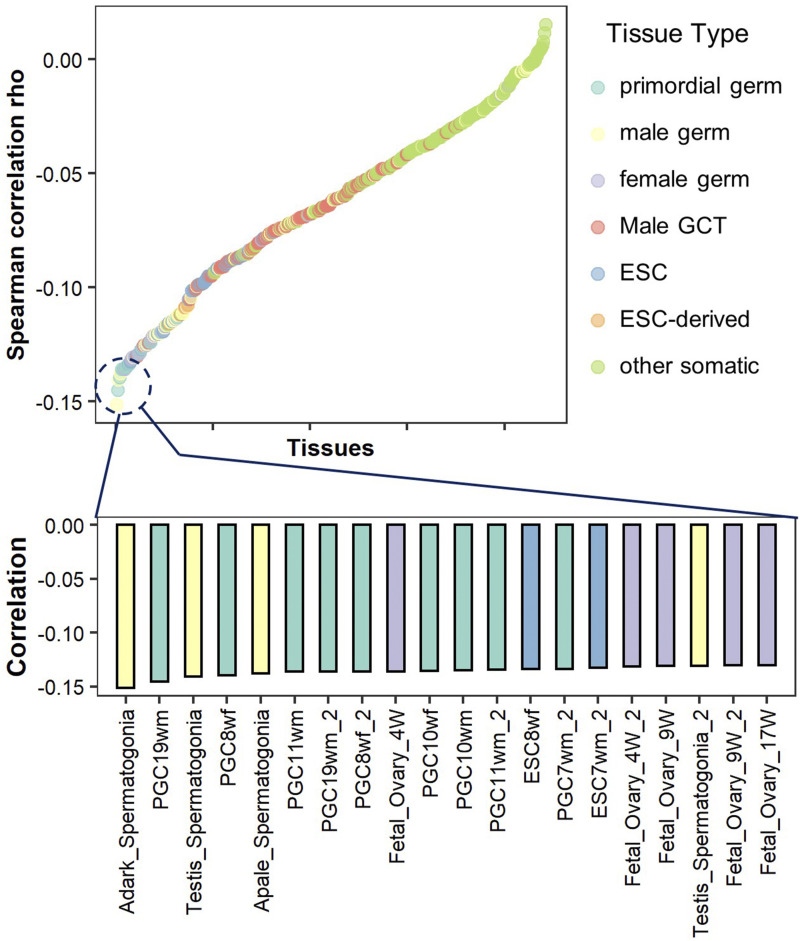
Spearman correlations between gene expression and the CpG_O/E_ ratio in the promoter regions with high tissue differentiation. Each of the 442 tissue samples is represented by a single dot. The color indicates a specific tissue type. ESC: embryonic stem cells. GCT: germ cell tumors. Dots are ordered from left to right by the correlation (rho value).

As can be seen in Figure 4, 18 out of the lowest 20 significant correlation of expression and mutation are of germline (bottom histogram). This indicated a more significant correlation of methylation and CpG mutation rate since methylation negatively correlates with expression. At a broader scale in the top figure, we observed the expression of germ tissues, including primordial germ, male germ and female germ, negatively correlated with mutation rate while expression of other somatic tissues barely correlated with mutation rate.

We also used CpG_O/E_ in the promoter region as a measure of mutation rates, which reflected the accumulation of heritable CpG mutations over evolutionary time and negatively correlated with the germline mutation rate at CpG sites. The Spearman correlation between CpG_O/E_ and the gene expression level was computed for all tissues. As shown in Figure S3, gene expression correlated more significantly with CpG_O/E_ in most germline tissues than it did in other tissues. Remarkably, we found that the correlation was strongest in primordial germ tissues and was followed by the male germline and female germline. We barely observed correlation among most somatic tissues in Figure S3. The significant correlation between expression and CpG_O/E_ is indirect evidence of the strong positive correlation between the methylation level and the germline CpG mutation rate in the promoter region of primordial germ tissues.

These two results indicated that CpG mutation in promoter region correlated more with methylation in germline tissues than somatic tissues. One possible explanation is that the mutations in germ relevant tissues are more likely to accumulate in generations. The methylome of gamete genesis may have an impact on germline mutations, but now methylome data for these stages is not available. Obtaining methylome data of these stages in the future may expand our vision in the methylation impact on germline mutation.

## Discussion

In this paper, we investigated the relationship between dynamic methylation levels and germline CpG mutation rates across 13 developmental stages. At the single-site level, we discovered a significant correlation between the DNA methylation level and the rate of point mutations at CpG sites. The regional methylation level was significantly correlated with the CpG C > T mutation rate among most genomic regions and chromosomes.

The impact of methylation on germline mutation has long been a focus topic for scientists. Interestingly, our result is different from 2 previous researches (Xia *et al.* 2012; Kusmartsev *et al.* 2020) which reported few correlation between methylation and mutation. These two researches applied methylome of the ^1^H embryonic stem cell which is somatic instead of germline-relevant. Xia *et al.* used human-chimpanzee divergence as a proxy for germline mutations. We suppose the differences in results stem from the different data choices.

We expected to identify a high level of correlation between the methylation level and C > T CpG mutation rate in the intergenic region, which is under neutral evolution. Regional analysis of 1 kb windows in intergenic regions showed a low correlation between methylation and the CpG C > T mutation rate in most cell stages (Figure 4D). We think that there are many repeat elements in the intergenic region, leading to difficulty in alignment. The SNP calling process was then affected by low-quality alignment, which in turn resulted in an inaccurate mutation rate in the intergenic region.

Although we have illustrated dynamic correlations for 13 stages, one limitation of our research is lacking DNA methylome in other germline development stages. Because of restrictions in technology and ethics for human samples, we could hardly obtain whole-genome methylome during spermatogenesis or oogenesis stages. With the rapid development in single-cell sequencing technology, we believe methylome in those missing stages would be available soon. Methylome in more germline development stages could help us reveal a broader impact of methylation on germline development and germline mutations.

Another limitation is that we only focused on changes in autosomes and differences caused by sex effect has not been taken into consideration. The processes of male and female germline development vary a lot, so the researches for two sexes should be separately designed. The male germline development involves spermatogenesis where paternal age should be considered. Our manuscript applied rare polymorphisms from large scale population analysis, so fathers’ age is hardly estimated. On the other hand, female germline development involves the X-chromosome inactivation. The X chromosome inactivation is scarcely studied under current bisulfite sequencing technology.

To understand the mechanism of methylation-associated mutability, further investigations of the functions of DNA repair systems need to be considered. However, regardless of the precise mechanism, our study provides strong evidence that site-level methylation plays an important role in CpG mutations in the germline across human developmental stages. Our study might help unravel the contribution of DNA methylation in shaping the emergence of germline variants at different genomic scales and different developmental stages, which will also provide information regarding the inheritance of epigenetic features and inherited diseases.
